# Effect of photon radiotherapy on microleakage and microhardness of nanohybrid resin composite restoration: an in vitro study

**DOI:** 10.1186/s12903-026-09873-4

**Published:** 2026-10-08

**Authors:** Manar Ahmed Khedr, Yasser Fathy Hussien, Mostafa A. Hashem, Reem Gamal

**Affiliations:** 1 Biomaterials Department, Faculty of Dentistry, Assiut University, Assiut, Egypt; 2 Biomaterials Department, Faculty of Dentistry, Minia University, Minia, 61511 Egypt; 3 South Egypt Cancer Institute, Radiation Oncology and Nuclear Medicine Department, Assiut University, Assiut, Egypt

**Keywords:** Microleakage, Vickers microhardness, Marginal integrity, Nanohybrid Resin composite, Photon Radiotherapy

## Abstract

**Background:**

Photon radiotherapy (RT) is widely used in the management of head and neck cancer (HNC) and may affect dental restorative materials. However, limited evidence is available regarding its effects on nanohybrid resin composite (RC) restorations. This research aimed to assess the impact of photon radiation treatment on microleakage (ML) and microhardness (MH) of nanohybrid RC restorations.

**Methods:**

A total of 54 samples were randomly divided into three groups (18 sample each): a control group (non-irradiated) and two RT groups exposed to 50 Gy (Gy) and 70 Gy of photon radiation. The radiation doses were delivered as a single dose, and post-irradiation assessments were performed immediately after irradiation. For ML, 30 extracted bovine incisors (*n* = 10 per group) were standardized, prepared with Class V cavities, and restored using a nanohybrid RC (Grandio, VOCO GmbH, Germany). Microleakage was assessed by dye penetration at the restoration interface using a stereomicroscope. For MH, 24 disc shaped composite samples (*n* = 8 per group) were evaluated using the Vickers MH test.

**Results:**

The median ML scores were 1.0, 1.0, and 2.0 for groups I, II, III, respectively (*p* = 0.029), while the median ML percentages were 17.93%, 24.75%, and 46.71%, respectively (*p* = 0.003). Before irradiation, mean MH values were 52.39 ± 2.41, 52.53 ± 1.51, and 52.23 ± 2.03, respectively (*p* = 0.959). Immediately after irradiation, these values increased to 53.60 ± 1.09, 62.90 ± 3.09, and 72.20 ± 5.53, respectively (*p* < 0.001).

**Conclusions:**

Under the conditions of this in vitro study, photon RT was associated with increased ML at 70 Gy of nanohybrid RC, whereas MH significantly increased following irradiation at both 50 and 70 Gy.

## Introduction

Head and neck cancer (HNC) ranks among the most frequent malignancies globally, constituting the seventh most frequently diagnosed cancer, with over 500,000 new cases diagnosed each year [1, 2]. Radiotherapy (RT) remains a main approach in the management of HNC, utilized either as a primary treatment or alongside surgery and chemotherapy [3, 4]. Photon-based RT, in particular, exerts its therapeutic effect by inducing DNA damage in cancer cells, thereby inhibiting their proliferation and survival. Given the anatomical complexity of the head and neck region, RT is especially valuable for treating malignancies of the oral cavity, pharynx, larynx, and salivary glands, contributing significantly to local tumor control and improved patient outcomes [1, 5, 6]. The RT for HNC is typically delivered in fractionated doses over several weeks, with cumulative radiation doses ranging from approximately 50 and 70 Gy (Gy) [2, 3]. Therefore, 50 and 70 Gy were selected in the present study to represent cumulative radiation doses within the clinically relevant range commonly used in head and neck RT. While this approach enhances tumoricidal effects and permits normal tissue recovery between sessions, it also poses risks to adjacent healthy structures, including oral tissues and dental restorations [1, 7]. 

Xerostomia, mucositis, dysgeusia, and dysphagia are common adverse effects of head and neck RT that can markedly impair patients’ quality of life [2, 8]. The RT can also damage dental tissues, affecting enamel, dentin, the dentin-enamel junction, and pulp [9]. Reduced salivary flow, microbial changes, poor oral hygiene, and radiation exposure increase the risk of extensive caries, while radiation-induced alterations in enamel and dentin mineral structure can reduce their acid resistance, hardness, and strength [10]. Therefore, careful selection of restorative materials is essential to ensure that their properties and sealability remain stable under irradiation [11, 12]. 

In addition to its damaging effects on healthy oral tissues, research has also shown that RT may modify the characteristics of restorative materials [13–15]. Among these, resin composites (RC) are commonly used in restorative dentistry due to their aesthetic appearance and effective bonding to tooth substrates [16, 17]. However, exposure to ionizing radiation may induce structural and chemical alterations in these materials, potentially compromising their mechanical integrity and clinical longevity [7]. The RC is a polymeric material typically composed of a methacrylate-based organic matrix incorporating inorganic fillers within the resin, along with initiators and catalysts [16, 18]. A previous study reported that RT energy can break specific chemical bonds within polymerized materials. However, at the same time, it can induce post-cure reactions in methacrylate monomers, altering their mechanical characteristics [19]. However, other studies contradicted this finding, reporting that RT has no impact on the polymerization or mechanical properties of these materials [20–24]. 

Microleakage (ML) is an important indicator of the interfacial sealing ability of restorative materials, as marginal leakage may contribute to marginal staining, sensitivity, recurrent caries, and eventual restoration failure [25]. Microhardness (MH) is a useful indicator of the surface mechanical properties of restorative materials and may provide information regarding their wear resistance and surface durability [26]. Therefore, evaluating both ML and MH provides complementary information regarding the interfacial sealing and surface mechanical performance of RC restorations following RT.

The effect of RT on the properties of RC or on its bonding to tooth structure remains controversial. Previous investigations have reported inconsistent findings regarding the effects of RT on the ML and MH of RC. These discrepancies may be related to differences in RC composition, radiation dose, irradiation protocols, and testing methodologies. In addition, relatively limited evidence has addressed the effects of cumulative radiation doses on both the mechanical and sealing properties of nanohybrid RC within the same experimental setting. Therefore, further investigation is needed to clarify the response of these materials to different levels of photon irradiation. The present study addresses this gap by evaluating both ML and MH of a nanohybrid RC following exposure to 50 and 70 Gy of photon irradiation. Understanding these effects may help inform the selection and evaluation of RC restorative materials for patients undergoing head and neck photon RT. So, this study aimed to evaluate the impact of different radiation doses on ML and MH of nanohybrid RC. The null hypothesis stated that photon irradiation does not influence the ML or MH of the evaluated RC material.

## Materials and methods

The study received ethical clearance from the Research Ethics Committee at the Faculty of Dentistry, Minia University (Approval No. 824), and all ethical guidelines for in vitro research were followed.

### Sample size calculation

Sample size determination was performed a priori using G*Power software (version 3.1.9.4) by selecting F tests → ANOVA: Fixed effects, omnibus, one-way, with α = 0.05 and a statistical power of 80%. Calculations were based on the between-group comparisons considering three independent groups for both tests. For the ML assessment, an effect size (f) of 0.6 was calculated considering the reported finding in the previous studies of Jornet et al. [27] and de Vasconcelos et al. [7] who demonstrated increased ML in irradiated compared with non-irradiated RC restorations. In their in-vitro study, resulting in a total required sample size of 30 samples (*n* = 10 per group). For the MH evaluation, an effect size (f) of 0.69 was calculated from the group means and standard deviation reported by Lima et al. [6], leading to a required total sample size of 24 samples (*n* = 8 per group).”

### Study design

A total of 54 samples were fabricated following the manufacturer’s instructions. Then, they were allocated into three primary groups (*n* = 18 each) in light of the radiation dose received: Group I (control, non-irradiated), Group II (subjected to 50 Gy), and Group III (subjected to 70 Gy). Each group was further distributed according to the test type, with *n* = 10 samples allocated to the ML test and *n* = 8 samples allocated to MH test.

### Samples preparation

For ML testing, 30 sound bovine incisors, free of caries and cracks and of approximately the same size, were obtained from the Faculty of Veterinary Medicine, Assiut University. Bovine teeth were selected as an alternative to human teeth in the present in vitro study because of their compositional and structural similarities to human teeth. After cleaning and storage in 0.1% thymol solution at 4 °C for no more than one month, Class V standard cavities (3 mm in diameter, 2 mm in depth) were prepared on the cervical third of the labial surface using a high-speed round diamond bur by a single operator. A new diamond bur was used for each cavity preparation to minimize variations in cutting efficiency associated with bur wear and to ensure uniform cavity preparation conditions. This was followed by finishing with a yellow-coded diamond stone. Cavity dimensions were verified with a periodontal probe. Enamel was selectively etched for 15 s using Meta Biomed etchant (Korea), rinsed, and gently blotted. A universal adhesive (Bisco, INC., 1100 W. Irving Park Rd., Schaumburg, IL 60193, USA) was applied in two coats, each air-thinned, and then light-cured for 20 s as per the manufacturer’s recommendations using an LED light-curing unit (RTA MiniS, Guilin Woodpecker Medical Instrument Co., China; intensity: 1000 mW/cm²). The intensity of the output light was measured and calibrated using a radiometer (LM-100; DigiRate). Cavities were filled in layers with the nanohybrid RC (Grandio, VOCO GmbH, Germany). The light-guide tip was positioned in direct contact with the Mylar strip covering the RC surface during photopolymerization, corresponding to a nominal distance of 0 mm.

To prepare for dye penetration testing, the teeth had their apices sealed using dental wax, and the coronal portion had two layers of nail polish applied to it, leaving a 1 mm margin around the restoration exposed. All cavity preparations and RC restorations were performed by a single operator to minimize operator-related variability and ensure consistency throughout the experimental procedure. Then, the 30 samples were numbered from 1 to 30 and randomly allocated to the three experimental groups (*n* = 10/group). The allocation was performed manually by an individual who was not involved in the cavity preparation or restoration procedures to minimize potential selection bias.

Twenty four disc shaped samples (*n* = 8 per group) were prepared from the nanohybrid RC for MH testing using Teflon molds measuring 15 mm in diameter and 1 mm in height. The molds were placed on a glass slab and packed with the RC. Following the manufacturer’s guidelines, light curing was performed for 20 s. To ensure a smooth, flat, and void-free surface, the filled mold was covered with a glass slab, and a 500 g weight was applied for 1 min prior to curing. Curing was then performed through the upper glass slab. After curing, the samples were finished and polished as directed by the manufacturer and subsequently stored in distilled water for 24 h.

### Radiation exposure

Samples of groups II and III were taken out of distilled water, dried, and then positioned in wax blocks for irradiation. The ML samples were placed in a cavity (1 cm deep) within a 20 × 20 × 6 cm³ block made of pink modeling wax (Tenatex eco toughened dental modelling wax, Associated Dental Products Ltd, Kemdent Works, Purton, Swindon, Wiltshire, SN5 4HT, United Kingdom) and covered with an additional 1.4 cm thick wax layer to ensure adequate radiation buildup. Water was used to fill any gaps between the samples to eliminate air spaces and maintain sample hydration during irradiation. Irradiation was performed under stable room-temperature conditions, with no noticeable change in ambient temperature during the procedure. For the MH samples, a smaller wax block was used (10 × 10 × 6 cm³), with a cavity depth of 0.4 cm and a wax cover layer of 1.3 cm thickness.

Radiation exposure was performed using a digital linear accelerator (Elekta Synergy Platform digital linear accelerator, Elekta, Sweden) at Department of Radiation Oncology and Nuclear Medicine, South Egypt Cancer Institute, Assiut University, Assiut, Egypt. The beam energy was 6 MV, the source-to-surface distance (SSD) was 98.5 cm at dmax, and the source-to-axis distance (SAD) was 100 cm. The radiation field size was set to 20 × 20 cm² for ML samples and 10 × 10 cm² for MH samples. Group II and III samples received a single dose of exposure of 50 Gy and 70 Gy, respectively. The delivered doses were calculated using treatment planning system (MONACO 5.11). It was then verified using an ionization chamber according to IAEA TRS-398 “Absorbed Dose Determination in External Beam Radiotherapy” protocol [28]. The measurements were performed under the same beam energy and relevant irradiation conditions used for sample irradiation. The measured dose was compared with the prescribed dose to confirm that the samples received the intended radiation dose. Although clinical RT is typically delivered in fractionated doses, a single dose irradiation protocol was used in the present in vitro study as an experimental design choice to investigate the effect of defined cumulative radiation doses on the restorative material. This approach provided a standardized and reproducible radiation exposure while minimizing variability associated with repeated sample repositioning, handling, and changes in sample condition. Furthermore, since the primary objective of clinical fractionation is to allow normal tissue recovery between sessions, fractionation was unnecessary in this experimental design. Meanwhile, Group I samples underwent the same sample handling, preparation, storage conditions, and duration but were not exposed to photon irradiation.

### Microleakage test

All restored teeth were dipped in a solution of 1% methylene blue dye (Pharma Technology Co., Ltd., India) for 24 h. Methylene blue was selected in the present study because of its strong staining ability and low molecular weight. After dye immersion, the teeth were rinsed under running water and embedded in acrylic resin blocks measuring 50 mm long by 30 mm wide by 10 mm high. Each tooth was then split in a buccolingual direction through the midpoint of the restoration using a linear precision saw (IsoMet 4000 Linear Precision Saw, Buehler, USA). Following sectioning, the mesial half of each tooth was selected for ML evaluation. The ML was assessed using two methods: a scoring system and the percentage of ML. Dye penetration along the restoration margins was assessed with a stereomicroscope (OLYMPUS SZX16, Japan) at 15× magnification. No cyclic mechanical loading simulation was performed before the ML assessment.

**Fig. 1:**
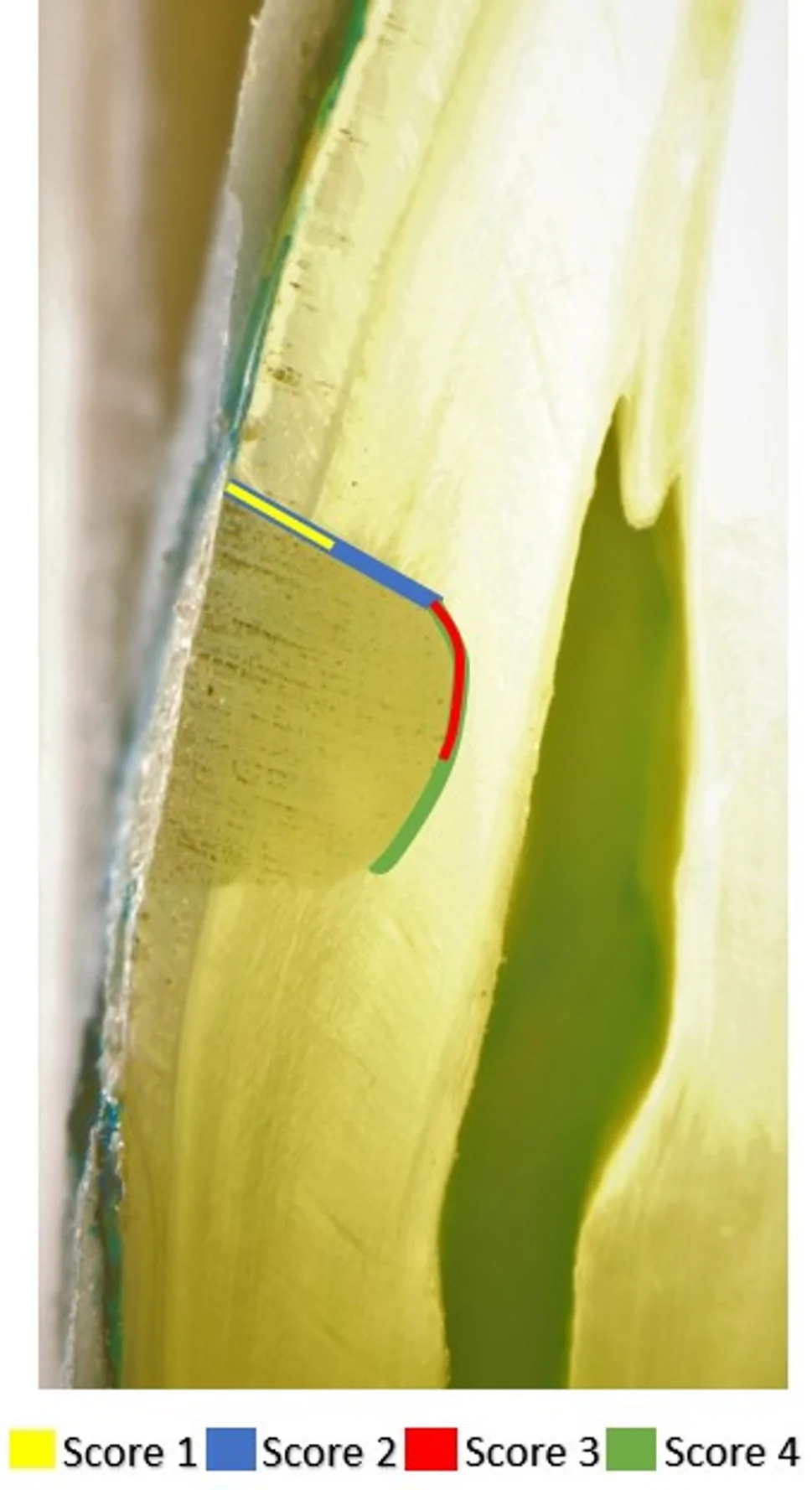
Illustration demonstrating microleakage scores

The ML was scored following the protocol outlined by Didron et al. [29] as follows (Fig. 1):

- 0 = dye penetration is absent.
- 1 = dye penetration up to half the cavity wall.
- 2 = penetration along the entire cavity wall.
- 3 = penetration up to half the axial wall.
- 4 = penetration beyond half of the axial wall.

Dye penetration depth and total cavity wall length were quantified by ImageJ analysis software (ImageJ 1.54 g; Java 1.8.0_345 [64-bit], US). The ML was then expressed as a percentage by calculating the ratio of the dye penetration length to the total cavity wall length and multiplying the result by 100. The combined use of both methods provided complementary qualitative and quantitative assessments of ML. The ML evaluator was blinded to the group allocation during the assessment to minimize the risk of observational bias. The ML scores and ImageJ measurements were performed by the same examiner. To assess intra-examiner consistency, a subset of samples was reassessed and remeasured by the same examiner.

### Microhardness test

The MH was measured using a Vickers MH tester (Wilson Hardness, Buehler ITW Test & Measurement GmbH, Germany). A diamond indenter was applied to the sample surface with a 50 g load and a dwell time of 15 s. Each sample was tested two time points: before and after irradiation. Initial measurements (before irradiation) were performed for all groups 24 h after sample preparation. Following the initial measurements, group I samples were returned to distilled water and maintained under the same storage conditions and for the same duration as the irradiated groups until completion of the irradiation procedure. During irradiation, the samples of groups II and III were maintained in the wax blocks with water filling the spaces between them and maintaining hydration, as described above. Immediately after irradiation, all samples were removed from the water and subjected to the final MH measurements (after irradiation) at room temperature. For the before and after irradiation measurements, the surface of each sample was divided into three predefined areas. One indentation was made in each area before irradiation, and a separate indentation was made at a different location within the corresponding area after irradiation. Thus, a total of six indentations were made per sample: three indentations before and three indentations after irradiation to obtain a representative estimate of MH while minimizing the potential influence of local material heterogeneity and indentation interaction. All indentations were positioned at least 1 mm apart from one another and at least 1 mm from the sample edges to minimize any potential influence of adjacent indentations or edge effects. The average of the three indentations was calculated to determine the Vickers Hardness Number (VHN). The testing procedure was performed in accordance with the methodology described by Lima et al. [6], with the number of indentations adapted to three indentations per specimen at each measurement time. The Vickers MH tester was calibrated before the measurements according to the manufacturer’s recommendations. Measurement consistency was checked by repeating the measurements under the same testing conditions.

### Statistical analysis of the data

The data was input into a computer, and statistical analysis was performed utilizing IBM SPSS software (version 27.0; IBM Corp., Armonk, NY, 2020). Using the Shapiro-Wilk test, continuous variables were evaluated for normal distribution. For normally distributed quantitative variables, quantitative data were presented as the minimum–maximum range and mean ± standard deviation (SD). To compare three groups, the one-way ANOVA test was used, followed by the post hoc test (Tukey) for pairwise comparison. When the assumption of equal variances was not met (according to Levene’s test), Welch’s ANOVA was used, followed by the Games–Howell post hoc test for pairwise comparisons. A paired t-test was also performed to compare outcomes before and after. For quantitative variables that did not exhibit a normal distribution, data were summarized as the minimum–maximum range and median with interquartile range (IQR). Differences among the three groups were analyzed using the Kruskal–Wallis test, followed by Dunn’s post hoc test for pairwise comparisons. Effect size between independent groups was calculated using eta squared (η²) with 95% confidence intervals, while Cohen’s d was used for paired data. The statistical significance of the results was evaluated at a 5% significance level.

## Results

### Microleakage (ML)

**Fig. 2:**
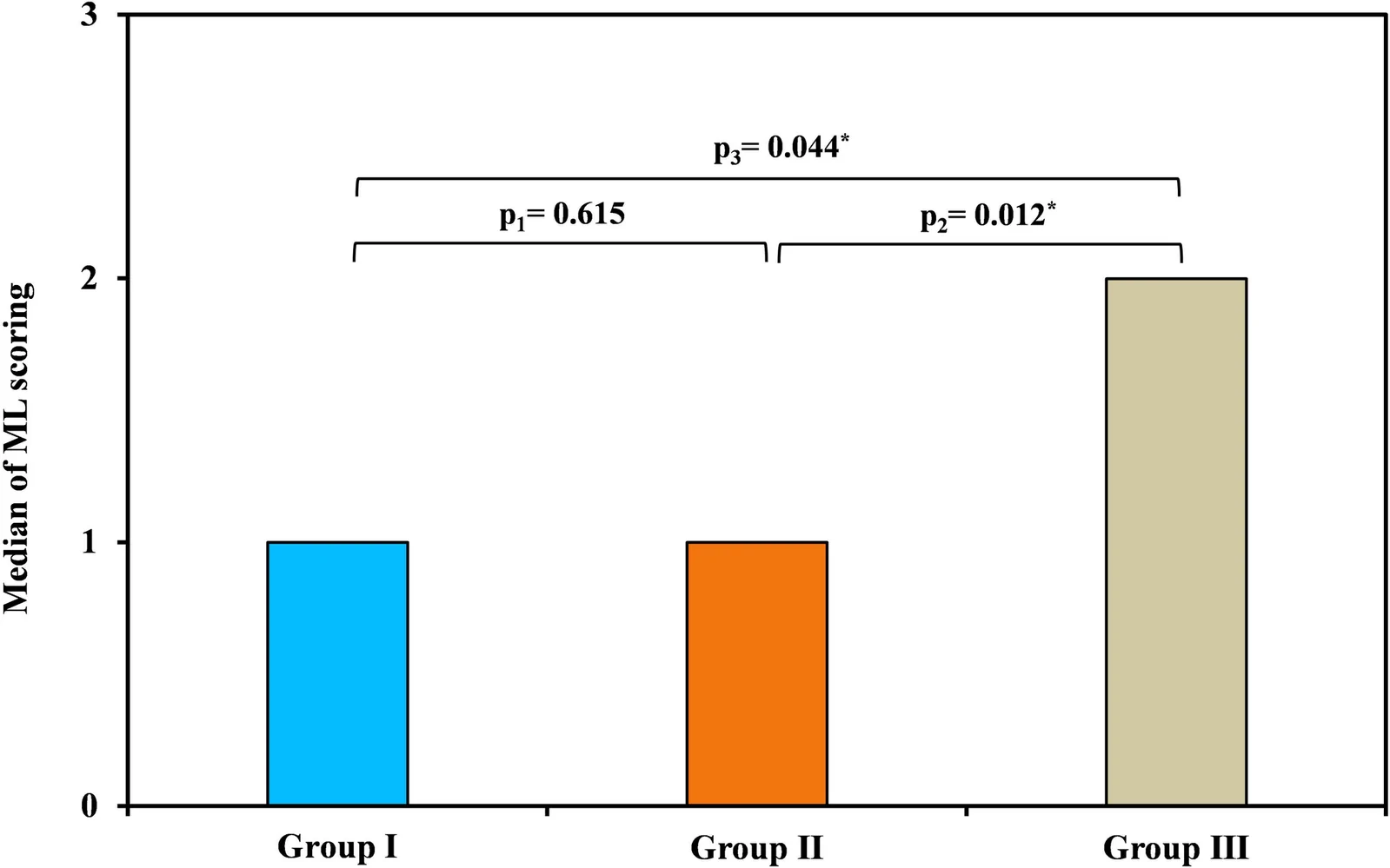
Bar chart illustrates comparison of the three groups under study based on ML scoring

The medians of ML scores for the three groups differed significantly (*p* = 0.029, η² = 0.189). Pairwise comparisons showed significant differences between Group I and Group III (*p* = 0.012) and between Group II and Group III (*p* = 0.044), whereas no significant difference was observed between Group I and Group II (*p* = 0.615) (Table 1; Fig. 2).

**Table 1:** Comparison of the three examined groups according to ML score and ML %

Groups	Group I (*n* = 10)	Group Ⅱ (*n* = 10)	Group Ⅲ (*n* = 10)	H	*p*	Effect Size
						η^2^ (95% C.I)
ML scoring						
Min. – Max.	1.0–2.0	1.0–3.0	1.0–4.0	7.092^*^	0.029^*^	0.189 (0.030–0.667)
Median (IQR)	1.0^b^ (1.0 − 1.0)	1.0^b^ (1.0 − 1.0)	2.0^a^ (1.0 − 4.0)			
Sig. bet. grps	p_1_=0.615, p_2_ = 0.012^*^, p_3_ = 0.044^*^					
ML %						
Min. – Max.	3.10–49.65	8.59–43.42	18.16 − 100.0	11.982^*^	0.003^*^	0.370 (0.129–0.858)
Median (IQR)	17.93^b^ (6.08–24.77)	24.75^b^ (15.86–27.56)	46.71^a^ (28.94–100.0)			
Sig. bet. grps.	p_1_=0.279, p_2_ = 0.001^*^, p_3_ = 0.019^*^					

*IQR* Inter quartile range, *SD* Standard deviation, *η*^2^ Eta Square, *CI* Confidence interval, *LL* Lower limit, *UL* Upper Limit

H: H for Kruskal–Wallis test. Pairwise comparisons between each two groups were performed using Dunn’s post hoc test for multiple comparisons

p: p-value for comparing the three groups under study

p_1_: p-value for comparing Groups I and II

p_2_: p-value for comparing Groups I and III

p_3_: p-value for comparing Group II and III

*Statistically significant at *p* ≤ 0.05

Medians in the same Row with any Small Common letter ^(a-b)^ are not significant (OR Medians with totally different letters ^(a-b)^ are significant)

**Fig. 3:**
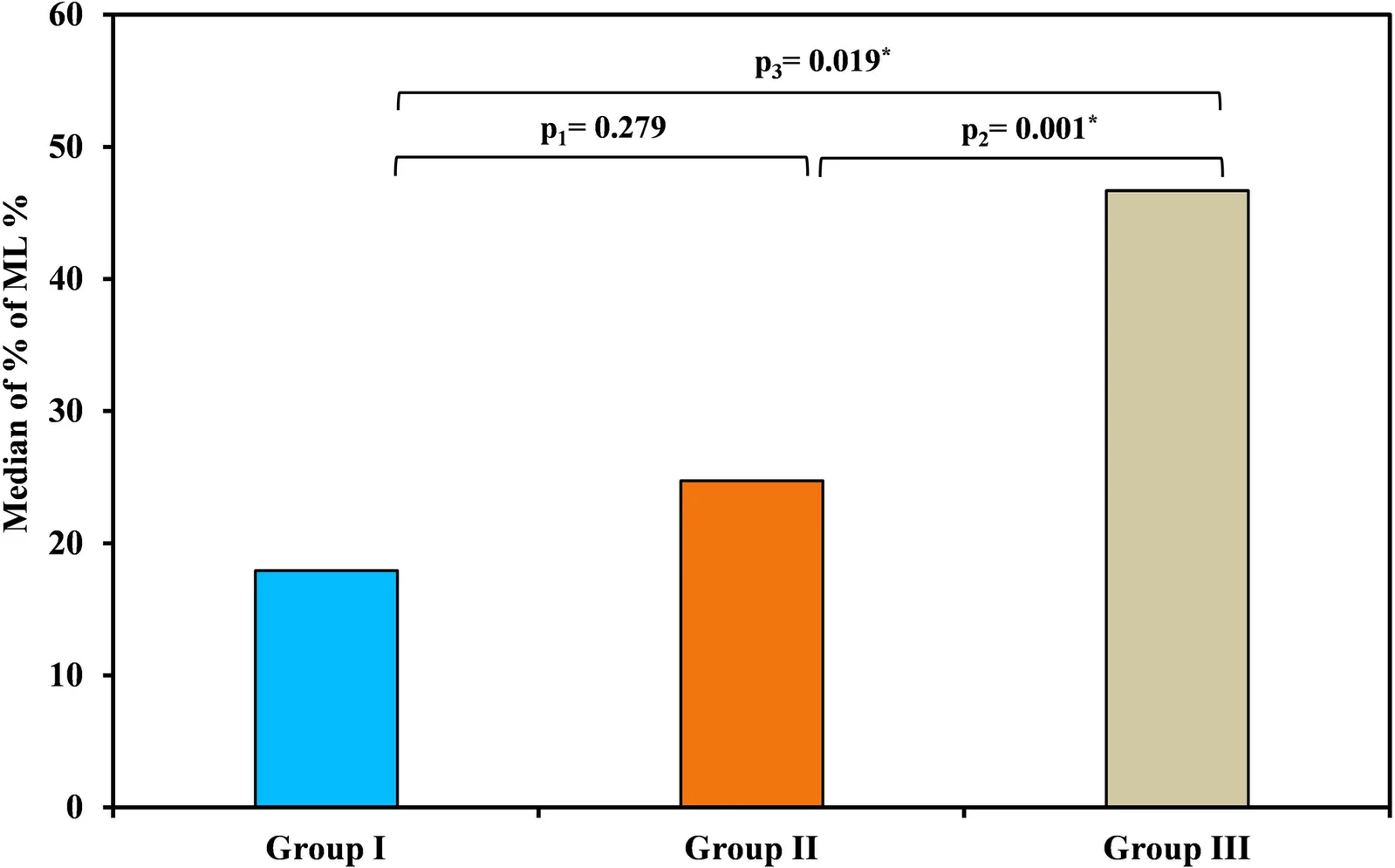
Bar chart illustrates comparison of the three groups under study based on ML%

The means of ML values differed significantly (*p* = 0.003, η² = 0.370). Significant differences between Groups I and III (*p* = 0.001) and between Groups II and III (*p* = 0.019), whereas no significant difference was observed between Groups I and II (*p* = 0.279) (Table 1; Fig. 3).

### Vickers microhardness

**Fig. 4:**
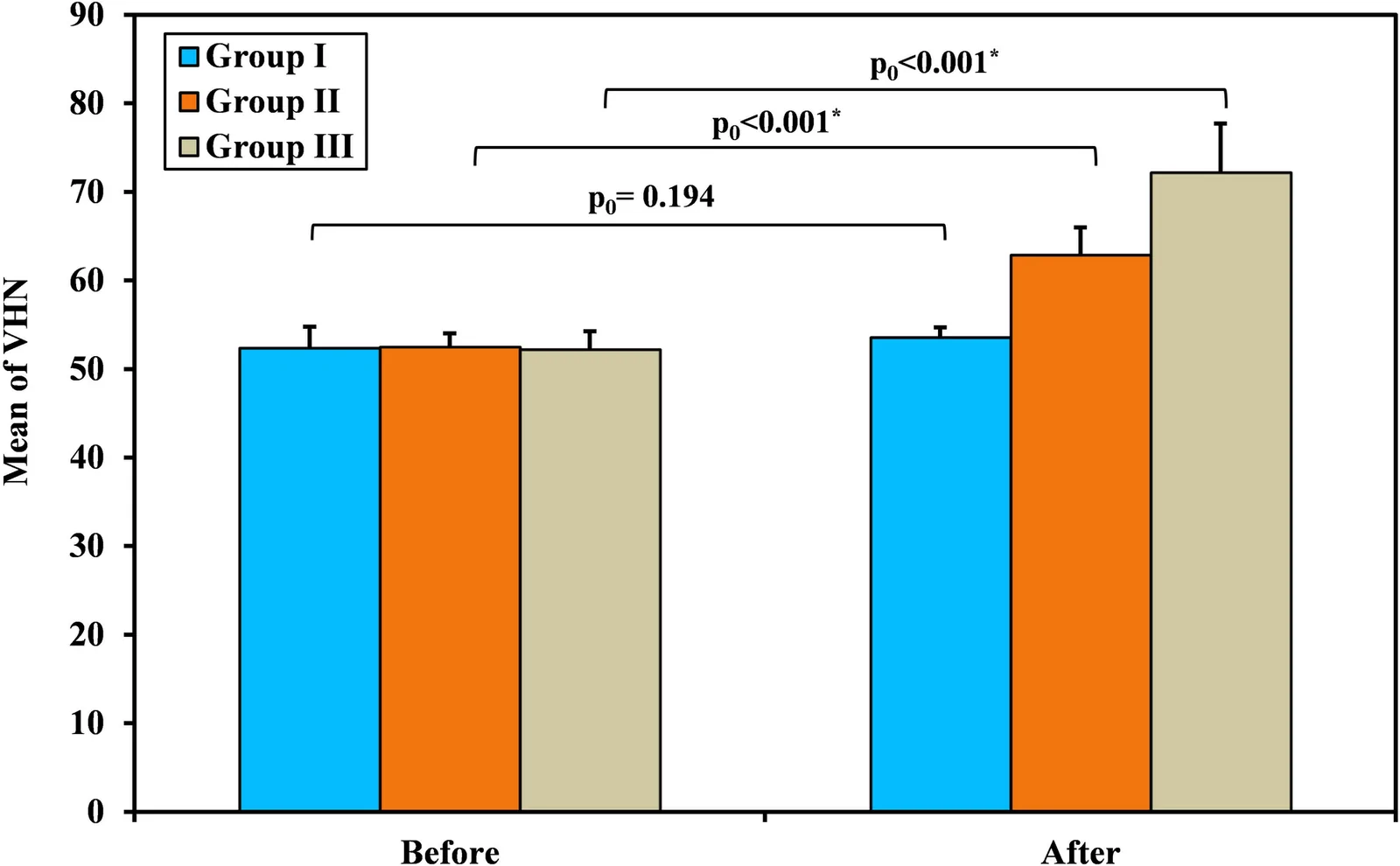
Bar chart illustrating the mean VHN of the different groups before and after irradiation

Before irradiation, no significant difference was observed among the three groups (*p* = 0.959, η² = 0.004) (Table 2; Fig. 4). After irradiation, a significant difference was observed among the three groups (*p* < 0.001, η² = 0.827). Pairwise comparisons showed significant differences between all groups (Group I vs. Group II, Group I vs. Group III, and Group II vs. Group III; *p* < 0.001). Group III showed the highest post-irradiation VHN, whereas Group I showed the lowest. Within-group comparisons showed that VHN did not change significantly in Group I (*p* = 0.194), whereas significant increases were observed in Groups II and III (*p* < 0.001 for both) (Table 2; Fig. 4).

**Table 2:** Min-Max values, means and SD. of VHN of different groups

Groups	Group I (*n* = 8)	Group Ⅱ (*n* = 8)	Group Ⅲ (*n* = 8)	Test of Sig.	*P*	Effect Size
						η^2^ (95% C.I)
Before						
Min. – Max.	48.83–55.63	49.70–54.77	48.40–55.0	F = 0.042	0.959	0.004 (0.000 − 0.040)
Mean ± SD.	52.39^a^ ± 2.41	52.53^a^ ± 1.51	52.23^a^ ± 2.03			
After						
Min. – Max.	51.17–54.70	58.57–66.93	64.63–78.03	^#^F = 67.091	< 0.001^*^	0.827 (0.625 − 0.884)
Mean ± SD.	53.60^c^ ± 1.09	62.90^b^ ± 3.09	72.20^a^ ± 5.53			
Sig. bet. grps.	p_1_<0.001^*^, p_2_ < 0.001^*^, p_3_ = 0.004^*^					
p_0_	0.194	< 0.001^*^	< 0.001^*^			
Cohen’s d 95% C.I	0.508 (-0.248–1.233)	3.106 (1.360–4.827)	3.623 (1.636 − 5.592)			

*SD* Standard deviation, *η*^2^ Eta Square, *CI* Confidence interval, *LL* Lower limit, *UL* Upper Limit

F: F for One way ANOVA test, Pairwise comparison bet. Each 2 groups was done using Post Hoc Test (Tukey)

^#^F: Welch ANOVA test, Pairwise comparison bet. Each 2 groups was done using Post Hoc Test (Games-Howell)

p: p value for comparing the three groups under the study

p_0_: p value for Paired t-test for comparing between before and after in each group

p_1_: p value for comparing Groups I and II

p_2_: p value for comparing Groups I and III

p_3_: p value for comparing Groups II and III

*Statistically significant at *p* ≤ 0.05

Means in the same Row with any Small Common letter ^(a−b)^ are not significant (OR Means with totally different letters ^(a−b)^ are significant)

## Discussion

The HNC is the seventh most frequently diagnosed cancer globally [30–32]. It can be treated with RT, either alone or in combination with surgery and chemotherapy [1, 33]. Photon RT remains more widely used than proton RT because of its lower cost and the limited evidence demonstrating clear superiority of proton RT [34]. Head and neck RT can adversely affect oral tissues and dental restorations, depending on factors such as radiation dose and the volume of exposed tissue [35, 36]. 

Patients receiving head and neck RT face a higher risk of dental disease, making the selection of appropriate restorative materials essential to prevent adverse interactions with radiation. Photon radiation can significantly modify the mechanical properties and durability of restorative materials, often indirectly through radiation-induced hyposalivation that damages the salivary glands [24, 37–39]. For restorations before or after RT in HNC patients, non-metallic, insoluble materials are preferred. The RC fulfill these requirements, offering excellent esthetics and a comparable elastic modulus to dentin and enamel, which helps distribute masticatory forces evenly [19]. 

Composite restorations are frequently indicated for anterior teeth and increasingly for posterior restorations. Advances in filler technology have reduced particle size from hybrid and micro-hybrid to nanoscale, improving polishability and gloss retention. Currently, the two main types of nanosized composites available are nano-filled and nanohybrid composites [18]. Currently, no single composite meets both the functional needs of posterior restorations and the aesthetic demands of anterior restorations. Nanohybrid composites, however, provide improved mechanical properties, with manufacturers claiming superior esthetics, strong durability, biocompatibility, and long-lasting performance [40]. 

Accordingly, this study was designed to examine the impact of various photon RT doses, frequently utilized in HNC treatment, on the ML and MH of nanohybrid RC restorations. Standard RT is typically delivered in 2 Gy fractions five times per week, with a cumulative dose ranging from 50 to 70 Gy to ensure local tumor control while reducing toxicity [1, 30, 31]. Accordingly, doses of 50 and 70 Gy were selected for this investigation. Since the study did not involve living tissues, the entire dose was delivered in a single exposure, thereby eliminating the need for fractionation and recovery periods.

Campos L. et al. reported that photon radiation effectively modifies the properties of polymer materials. The high energy of the radiation produces numerous excited molecules and ions along its path, initiating chemical reactions, including polymerization, grafting, crosslinking, and degradation [41]. 

The ML is an important concern in restorative dentistry because it reflects the integrity of the tooth–restoration interface and may contribute to marginal staining, sensitivity, recurrent caries, and restoration failure [25]. Among the available in vitro methods, dye penetration is commonly used because of its simplicity and reproducibility [25, 42, 43]. Class V cavities were selected in this study because they are simple to prepare and restore, reducing technique sensitivity [44]. 

The current study found that ML significantly increased with higher irradiation doses, consistent with previous findings [45–47]. This increase may be attributed to radiation induced hydrolysis, which can lead to adhesive resin degradation, weakened bonding, and disruption of the resin dentin interface, thereby compromising the hybrid layer [45]. Previous studies have also reported structural alterations in enamel and dentin following RT, including changes in hydroxyapatite crystals and lattice structure, with alterations observed from doses of 40 Gy and a reduced crystallinity index at higher doses (70 Gy), in addition to significant damage to the dentin collagen matrix at elevated radiation doses [46, 47]. These structural and interfacial changes may contribute to the increased ML observed with higher irradiation doses in the present study.

In contrast, previous studies reported that therapeutic radiation doses did not significantly affect the ML of RC restorations [48, 49]. Bulucu et al. [48] used a total radiation dose of 60 Gy with a microhybrid RC and performed thermocycling before dye immersion, while Seif [49] used 60 Gy of fractionated gamma radiation with a nano-filled RC. In comparison, the current study evaluated radiation doses of 50 and 70 Gy using a nanohybrid RC. These differences in radiation dose, RC composition, irradiation protocols, and thermocycling procedures may partly explain the discrepancy between the findings of previous studies and those of the current study.

The increased ML observed after photon irradiation may have important clinical implications for RC restorations in patients receiving head and neck RT. A compromised marginal seal may facilitate the penetration of oral fluids and bacterial products, potentially contributing to marginal discoloration, sensitivity, and secondary caries [50, 51]. This may be particularly relevant in irradiated patients, as radiation-induced salivary dysfunction increases susceptibility to dental caries and other oral complications [52]. Therefore, maintaining marginal integrity may be important for the longevity of restorations in this patient population. Previous studies reporting impaired marginal adaptation following irradiation further support the clinical relevance of this finding [36, 39, 45]. However, these implications should be interpreted cautiously because the present findings were obtained under in vitro conditions.

Mechanical properties are essential determinants of the longevity and clinical performance of restorative materials. The MH testing is commonly utilized to assess the surface mechanical characteristics of dental materials and hard tissues because of its simplicity, quick procedure, and minimal material requirement [26]. The MH is closely associated with wear resistance and surface roughness; softer materials usually exhibit lower wear resistance, making them more susceptible to discoloration, plaque accumulation, gingival irritation, and recurrent caries. Both Vickers and Knoop MH testers are effective for MH measurement. Nevertheless, the Vickers MH test remains the most frequently employed technique for evaluating MH in dental materials [53]. 

The current study revealed a statistically significant increase in VHN after irradiation. This finding is consistent with previous studies [5, 14, 22, 31], which reported improved mechanical properties of RC following photon irradiation. The observed increase may be related to the penetration of photon radiation into the resin matrix, where excitation and ionization generate reactive species that can promote additional interactions and crosslinking within the polymer network [5, 14, 22, 31]. Owing to the reduced mobility of molecular chains within the polymerized resin network, these reactive species may primarily form new bonds with adjacent groups, potentially improving mechanical properties without altering the degree of conversion. Therefore, the increased VHN observed in the present study may reflect radiation-induced changes within the resin matrix or at the resin filler interface rather than an increase in the degree of conversion. This interpretation is supported by a previous study investigating the same composite type, which reported no significant change in the degree of conversion after irradiation [54]. An alternative explanation is that photon irradiation may induce localized changes in the resin rich surface layer, resulting in greater exposure of inorganic filler particles. Since the surface MH of RC is strongly influenced by the amount and distribution of exposed filler particles, this radiation induced surface modification could contribute to the increased VHN observed in the present study. This mechanism has previously been proposed for RC exposed to therapeutic ionizing radiation and was supported by changes in surface micromorphology following irradiation [21]. 

Conversely, several studies reported no significant effect of irradiation on the MH of RC, in contrast to the findings of the present study [6, 19–21, 23, 24, 55]. Other studies reported a detrimental effect of therapeutic radiation on MH of RC [15, 56, 57]. 

These discrepancies may be attributed to differences in the composition and microstructure of the tested restorative materials, particularly the characteristics of the resin matrix and filler system [6, 15, 20, 55, 57]. The type, size, distribution, and proportion of filler particles may influence the material’s response to radiation and its resistance to radiation induced surface changes [20, 57]. Differences in radiation dose and irradiation protocol may also contribute to the variability among studies, as therapeutic radiation has been delivered using different total doses and fractionation protocols [6, 19, 20, 55]. Furthermore, variations in radiation source and energy, such as the use of Cobalt-60 or linear accelerators, as well as the timing of MH assessment, may affect the observed response. In addition, the high energy RT can influence the MH of RC in two opposite ways: by breaking bonds within the polymer network, leading to decreased MH, or by promoting crosslinking, which enhances MH. Thus, the balance between radiation induced structural modification and degradation may vary according to the restorative material and experimental conditions potentially explaining why previous studies have reported increased, decreased, or unchanged MH after irradiation [6, 15, 19, 20, 55, 57]. 

The findings of the current study led to the rejection of the null hypothesis for both tested properties. In addition, it is recommended to conduct further clinical studies to evaluate the influence of RT on the sealing ability and marginal integrity of RC restorations. It is also recommended to investigate different RC, adhesive systems, and restorative modifications that enhance resistance to radiation-induced degradation while maintaining or improving MH.

### Limitations of the study

This study has several limitations that should be considered when interpreting the findings. The investigation was conducted under in-vitro conditions; therefore, the results may not fully reflect the complex clinical environment. Furthermore, ML and MH were evaluated under laboratory conditions without cyclic mechanical loading, which was intentionally omitted to isolate the effect of radiation exposure and avoid introducing an additional experimental variable.

### Recommendations for future research

The present study evaluated ML and MH only; therefore, further investigations are needed to assess other relevant mechanical, chemical, and interfacial properties of nanohybrid RC following radiotherapy. Moreover, investigating leakage using human teeth is recommended using scanning electron microscopy (SEM) and energy-dispersive X-ray (EDX) analysis to evaluate the integrity of the hybrid layer. In addition, incorporating cyclic mechanical loading or other aging procedures is suggested, along with investigating different types of RC and adhesive systems to evaluate the long-term behavior regarding ML and MH of restorative materials following irradiation.

## Conclusion

Under the limitations of this in vitro study, photon RT was associated with increased ML of the nanohybrid RC restorations at 70 Gy. In contrast, MH showed a statistically significant increase following photon radiation exposure at both 50 and 70 Gy.

## Data Availability

The datasets used and/or analyzed during the current study are available from the corresponding author upon reasonable request.

## Abbreviations

RT: Radiotherapy
HNC: Head and neck cancer
RC: Resin composite
ML: Microleakage
MH: Microhardness
Gy: Gray
VHN: Vickers Hardness Number
SD: Standard deviation
IQR: Interquartile range
